# Omics in Myopia

**DOI:** 10.3390/jcm9113464

**Published:** 2020-10-28

**Authors:** Emil Tomasz Grochowski, Karolina Pietrowska, Tomasz Kowalczyk, Zofia Mariak, Adam Kretowski, Michal Ciborowski, Diana Anna Dmuchowska

**Affiliations:** 1 Department of Ophthalmology, Medical University of Bialystok, M. Sklodowskiej Curie 24a, 15-276 Bialystok, Poland; mariakzo@umb.edu.pl; 2 Clinical Research Centre, Medical University of Bialystok, M. Sklodowskiej Curie 24a, 15-276 Bialystok, Poland; kpietrowska89@gmail.com (K.P.); tomasz.kowalczyk000@gmail.com (T.K.); adam.kretowski@umb.edu.pl (A.K.); 3 Department of Endocrinology, Diabetology and Internal Medicine, Medical University of Bialystok, M. Sklodowskiej Curie 24a, 15-276 Bialystok, Poland

**Keywords:** myopia, metabolomics, proteomics, ocular biomarkers, mass spectrometry, liquid chromatography, gas chromatography, capillary electrophoresis

## Abstract

Myopia is a globally emerging issue, with multiple medical and socio-economic burdens and no well-established causal treatment thus far. A better insight into altered biochemical pathways and underlying pathogenesis might facilitate early diagnosis and treatment of myopia, ultimately leading to the development of more effective preventive and therapeutic measures. In this review, we summarize current data about the metabolomics and proteomics of myopia in humans and present various experimental approaches and animal models, along with their strengths and weaknesses. We also discuss the potential applicability of these findings to medical practice and suggest directions for future research.

## 1. Introduction

Myopia is a condition in which the spherical equivalent objective refractive error in either eye decreases to ≤–0.50 diopter (–0.50 D), whereas high myopia (HM) is defined as the spherical equivalent objective refractive error in either eye ≤–5.0 D (according to WHO) or ≤−6.0 D (according to other sources). HM is associated with a significant axial elongation of the eye, with >26.0 or 26.5 mm being the most common threshold. HM accompanied by sight-threatening macular pathologies, such as choroidal neovascularization, chorioretinal atrophy (diffuse or more severe) [1] or Fuch’s spot, can be classified as pathological myopia (PM) [2]. Myopia, especially HM, is associated with increased risk of non-macular conditions, such as rhegmatogenous retinal detachment (RRD), open-angle or pigmentary glaucoma, earlier cataract development and topical steroid hypersensitivity.

An increase in the prevalence of myopia has been observed globally, especially in East Asia, where this condition is found in up to 90% of school-leavers [3]. A plethora of factors, including ethnic/genetic susceptibility, environmental congestion, early education, near distance-related activities and reduced time outdoors, are suspected to increase the risk of myopia development [4]. The incidence of myopia varies significantly between populations, age groups and even people with various educational levels and has been steadily increasing over the last few decades. By 2050, the numbers of patients with myopia and HM are projected to reach 5 billion and 1 billion, respectively, making conditions from this spectrum a global pandemic [5].

Although myopia has been studied for decades, novel research methods may provide a better insight into the pathogenesis of this condition, which might eventually stimulate progress in its prevention and treatment. Progress in analytical methods and continuous improvement of computational capabilities allowed for the development of omics techniques. The use of the omics allows for a holistic approach to myopia pathogenesis. Proteomics and metabolomics are the last two components in the omics cascade, aimed at a global measurement of proteins and metabolites, respectively. Mass spectrometry (MS) is commonly applied in both approaches, facilitating detection of thousands of molecules in a small volume of biological material, which may lead to a better understanding of human physiology in health and disease [6]. Identification of potential novel biomarkers and disturbed metabolic pathways may contribute to a better understanding of myopia pathophysiology and may indicate novel therapeutic targets. It would also facilitate stratification of patients according to their risk profiles and earlier diagnosis. This could have direct translation into prognosis. Especially taking into account the fact that choroid neovascularization is treatable with anti-VEGF injections, and can have dramatic consequences when left untreated, the frequency of follow-up visits could be adjusted accordingly. As another example, one may speculate that timely identification of patients with high risk for rhegmatogenous retinal detachment may encourage 360-degree laser cerclage, which is not a standard procedure. In general, this could direct the treatment of myopia towards individualized medicine.

## 2. Materials and Methods

The PubMed database was searched for articles containing “myopia metabolomic*” and “myopia proteomic*” phrases, indexed until 12 September 2020. Review articles, original papers missing detailed information about sample treatment and analytical conditions, as well as those not related to myopia and omics (e.g., restricted only to ELISA), were not included in the review.

Eventually, after detailed inspection and removal of duplicates, 34 original articles were found eligible for the review.

MetaboAnalyst 4.0 software (www.metaboanalyst.ca) was used for pathway analysis.

## 3. Results

Twenty-six proteomics-based articles, including four pure data articles, are summarized in Table 1. Seven of these studies included human patients, and nineteen were conducted in various animal models. Most of them were untargeted analyses of various samples, vitreous humor, retina and aqueous humor being the most common. Some of these studies aimed at the creation of proteome libraries, while others compared different animal models. Human studies focused on proteome comparison of myopic eyes to either emmetropic eyes or with concomitant pathologies. Orthokeratology lens and atropine as treatment for myopia were also assessed from the proteomic point of view.

Eight metabolomics-based studies, most of them untargeted, among them seven involving human patients and only one carried in guinea pigs, are summarized in Table 2. In contrast to proteomic studies, the metabolomic studies focused mainly on serum analyses. From strictly eye-related samples, the aqueous humor was analyzed.

**Figure 1:**
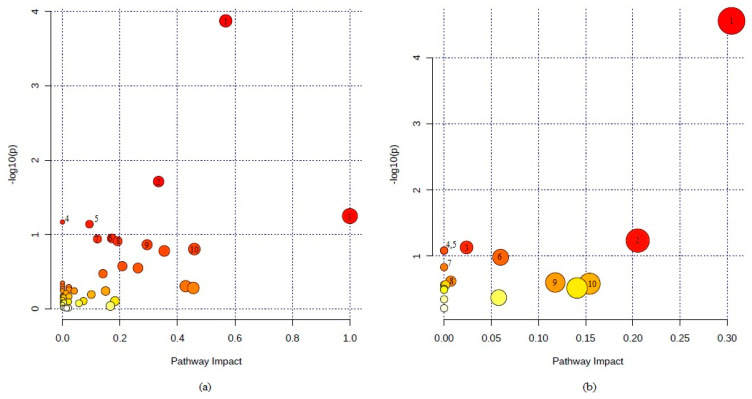
Metabolic pathway analysis performed for significant metabolites reported in human serum (**a**) and aqueous humor (AH) (**b**) samples. Metabolic pathway analysis was carried out with MetaboAnalyst 4.0 software. Ten most significant pathways identified for the serum samples: 1. sphingolipid metabolism, 2. citrate cycle (TCA cycle), 3. linoleic acid metabolism, 4. biosynthesis of unsaturated fatty acids, 5. alanine, aspartate and glutamate metabolism, 6. tryptophan metabolism, 7. glyoxylate and dicarboxylate metabolism, 8. tyrosine metabolism, 9. glycerolipid metabolism, and 10. retinol metabolism. Ten most significant pathways identified for the AH samples: 1. arginine biosynthesis, 2. alanine, aspartate and glutamate metabolism. 3. glyoxylate and dicarboxylate metabolism, 4. D-glutamine and D-glutamate metabolism, 5. nitrogen metabolism. 6. pyrimidine metabolism, 7. aminoacyl-tRNA biosynthesis, 8. pantothenate and CoA biosynthesis, 9. citrate cycle (TCA cycle), and 10. sphingolipid metabolism.

Inter-study pathway analysis was carried out separately for human serum and aqueous humor (AH) using MetaboAnalyst 4.0 software, as shown in Figure 1.

The pathway analysis carried out for 303 significant serum metabolites reported in five studies identified a total of 49 potentially involved metabolic pathways. As shown in Figure 1a, sphingolipid metabolism, as well as the citrate cycle (TCA cycle), were the most significantly affected metabolic pathways. In the case of AH samples, 29 metabolites reported in two studies corresponded to 18 potentially involved pathways. The most significant metabolic pathway identified in AH analysis (Figure 1b) was arginine biosynthesis, followed by alanine, aspartate and glutamate metabolism. A complete list of metabolic pathways involving metabolites identified in serum and AH samples is shown in Table 3 and Table 4, respectively. The tables contain the number of metabolites involved in a given pathway and detected in serum or AH, as well as the results of pathway analysis (*p*-value and pathway impact value).

Comparison of metabolic pathway analysis results for serum and AH samples identified 14 common pathways, mainly associated with decreased energy metabolism, increased oxidative stress, abnormal amino acid metabolism [37] or related to dopamine receptor D2 [35]. Metabolic pathways identified in the case of both serum and AH samples are highlighted in bold.

## 4. Discussion

### 4.1. Experimental Models

#### 4.1.1. Human Studies

Blood serum is the most accessible material for omics studies in humans. However, due to the influence of various mechanisms, such as the blood–retinal barrier, only a certain proportion of physiological processes taking place in the eye will be reflected in the systemic circulation. On the other hand, the availability of ocular tissue specimens from human patients is limited, as they are usually obtained during highly invasive ophthalmological procedures, cataract surgeries in the case of the aqueous humor (AH) [41,42] and pars plana vitrectomies (PPV) for the vitreous humor (VH). VH is considered a particularly valuable research material given its anatomical and functional link to the retina and choroid. Unfortunately, the fact that VH can be obtained solely during PPV limits its availability to patients with the most serious complications, such as RRD or myopic retinoschisis (MRS). For the same reason, controls in the research on myopia are usually recruited among patients qualified for PPV because of different, theoretically unrelated conditions, such as macular hole (MH), epiretinal membrane (ERM) and RRD. Such an approach not only precludes an unbiased comparison between the results of myopia patients and truly healthy controls but also confines the insight into the pathogenesis of ocular disease to its final stages, rather than providing information about the initial, crucial steps in the transition from the norm to pathology. Furthermore, it is impossible to determine whether the changes observed in the biological material occurred secondarily to the chronic disease or were directly related to its pathogenesis.

#### 4.1.2. Animal Models

A number of animal models have been developed to provide an insight into the pathogenesis of myopia, with the most widespread being form-deprived myopia (FDM) and lens-induced myopia (LIM). Both these models involve juvenile animals in which the processes of emmetropization are highly active and easy to disrupt. Emmetropization is a set of homeostatic control mechanisms that enable visual stimuli to be adequately focused on the retina. In brief, myopic defocus allows for hyperopic development, whereas hyperopic defocus is associated with myopic development [43]. Isolation of inputs from the higher brain by the blockage of ganglion cell signaling does not prevent the regulatory growth of the eye [44]. Optic nerve transection was shown to prevent myopia in LIM but not FDM, which implies that these two conditions may differ in terms of their underlying mechanisms [45]. On the other hand, parasympathectomy did not affect LIM development but prevented myopia in the FDM model [46]. While these findings might seem contradictory at first, they suggest that while some aspects of the eye growth regulation depend on the integration of visual stimuli within the central nervous system, at least some could occur independently at a local level.

In the FDM model, the treated eye is permanently covered with a translucent diffuser, while the other eye remains unobscured. Such treatment typically induces high myopia, with a decrease in spherical equivalent refraction (SER) down to −5.0 D or more depending on the species, albeit with substantial intersubject variability. However, the changes may also reflect the impact of light deprivation in the retina and do not necessarily correspond well with the mechanism involved in the development of myopia in humans. Nevertheless, the results of the FDM studies should not be neglected, as published evidence suggests that shorter time spent outdoors and disturbances in circadian rhythms may also contribute to myopia development [47,48].

In the LIM model, a negative power lens is placed before one eye of a juvenile animal, making it artificially hyperopic. This enforces emmetropization to refocus the light back on the retina, which is associated with rapid elongation of the affected eyeball. This condition simulates to a certain degree continuous near work of optic system in the human eye, perpetuated by growing educational pressure and popularization of smartphones and other hand-held devices even among the youngest children; this process is believed to contribute to an increase in myopia prevalence [49,50]. Differences and similarities between FDM and LIM, as well as inconsistent results obtained with the two models, are a matter of hot scientific debate, but this problem is beyond the scope of this review [46].

Various species have been used as the experimental models, among them guinea pig, chicks, tree shrew, mouse and tilapia. Among mammals, tree shrews are closely related to primates and have been considered as a surrogate for the latter in experimental studies.

Fox-Lrp2 deficient mice constitute an interesting model for processes being most likely responsible for the development of HM and PM. In one study involving this model, up to an eight-fold increase in the liquid vitreous fraction was observed in mutant mice when compared with the controls [15].

Chicks have been widely used as an animal model for myopia research; although the chick’s eye differs substantially from the human eye, other than scleral structures, certain factors affecting normal refractive development appear to be similar.

The lack of an animal model that accurately simulates human pathological myopic retinopathy with late pathological changes significantly hinders the research on the underlying mechanisms of PM.

### 4.2. Altered Pathways and Clinical Implications

#### 4.2.1. Proteomics

VH seems to be the best, relatively less invasively accessible surrogate material for research on human chorioretinal omics. In a study conducted by Wei et al. [10], patients subjected to PPV were classified based on the lack of PM signs (AL < 26.5 mm), presence thereof (26.5 mm < AL <29.0 mm) or occurrence of high PM (>29.0 mm), and then subdivided according to a specific pathology: MH, ERM, RRD or MRS. Expression of prostaglandin-H2 D-isomerase (PGDS) and glutathione peroxidase 3 (GPX3) in the PM groups was shown to be significantly lower than in the controls. The two enzymes are responsible for the scavenging of reactive oxygen species (ROS). The evidence from in vitro studies suggests that L-PGDS can potentially prevent oxidative stress and apoptosis-related neurodegenerative diseases. In turn, GPX3 is known to catalyze the reduction of organic hydroperoxides and hydrogen peroxide (H_2_O_2_) to protect cells from oxidative damage [10].

Some similarities in the composition of proteins from crystallin, keratin and cytoskeletal families were found in the AH of cataract surgery patients with a history of glaucoma, diabetes mellitus or HM, but not in the controls [19]. In another study, the AH from patients with HM was shown to contain twice the amount of vitamin D-binding protein and transthyretin than in emmetropic controls [28].

Atropine has shown some potential to inhibit myopia progression in several animal models and two clinical trials in humans [51,52]. However, the clinical application of 1% atropine is limited by its serious adverse effects, and recently, the interest of researchers worldwide has been shifted from high-concentration (1%) to low-concentration (0.01–0.05%) atropine. Unfortunately, 0.01% atropine seems only to slow down the refractive changes associated with myopia, without an effect on the axial elongation of the eye. Given that myopia-related pathologies are mostly associated with eye length, we still do not know whether atropine could be an effective treatment for HM. Moreover, it is unclear how exactly this muscarinic antagonist exerts its effect on the eye. As muscarinic receptors are localized throughout the neural retina, and significant amounts of them are present in the RPE, this might be a target. According to Barathi et al. [53], another putative mechanism of atropine action might be its effect at the level of GABA transporter 1 (GAT-1), as elevated concentrations of the latter in the myopic retina were shown to undergo a significant reduction in response to atropine treatment [20]. GABA is the major inhibitory neurotransmitter in the retina and other parts of the central nervous system, and GABA signaling to dopaminergic amacrine neurons was shown to be associated with the reduction of their burst activity [54,55]. Furthermore, atropine was recently found to bind to α_2A_-adrenergic receptors in amacrine cells as well. Meanwhile, α_2A_-adrenergic receptors are expressed on dopaminergic neurons, and both atropine and α_2A_-adrenoreceptor antagonists are known to stimulate dopamine (DA) release, whereas this process is strongly suppressed by α_2A_-adrenoreceptor agonists [56]. The impact of DA and melatonin (Mel) on myopia is discussed in detail in Section 4.2.2 below.

The articles presenting only proteomic findings, without the interpretation thereof, are of lesser value from a clinical perspective. However, they may constitute a reference for future research, serving as a proteome database for various biological materials, such as tears from patients wearing orthokeratological lenses as a treatment for myopia and normal controls [9], chick cornea in FDM [12], chick VH [8] and guinea pig retina [24] during the emmetropization period.

#### 4.2.2. Metabolomics

The authors of one study [40] stratified the results obtained with a guinea pig model for FDM according to a time point, as some of the animals were sacrificed on day 3 whereas others on day 14 since the FDM induction. In some guinea pigs, metabolite levels were significantly different from those found in the controls solely on day 3 (early responders), whereas in others, the significant differences were observed on both day 3 and 14 (continuous response) or solely on day 14 (late responders). In the early responders, mannose and glucose levels in FDM eyes were significantly higher than in control retinas, whereas concentrations of arabinose, urea, tyrosine and glutamic acid remained significantly lower. In the continuous response group, significantly lower levels of threonine, valine, isoleucine, alanine and malic acid were observed, and late responders presented with significantly lower concentrations of some fatty acids, namely arachidic, octadecenoic, octadecanoic, arachidonic (ARA), hexadecanoic, tetradecanoic and octadecadienoic acid. The observation that concentrations of some metabolites were significantly altered at some, albeit not all, points of the study constitutes another argument to support the hypothesis that research on subjects with already developed, “mature” myopia might not necessarily identify critical factors responsible for the development of this condition at its earlier stages.

Human AH has been a subject of metabolomics studies, both with GC-MS [34] and combined LC-MS, CE-MS [33] separation. In these studies, hundreds of significant associations were found on metabolite–metabolite correlation analysis; while the paucity of the data might hinder any definitive conclusions, it also adds considerably to our knowledge of human eye metabolome and its complexity.

The effects of two neurotransmitters, dopamine (DA) and melatonin (Mel), acting as mutual inhibitors in the regulation of circadian rhythms, on ocular diseases, especially myopia, have been studied in various animal models. To this date, the presence of specific DA and Mel receptors has been confirmed in frog [57,58], chick [59], guinea pig [60], mammalian [54,61] and human [62] retinal cells. Dopaminergic agents, administered either topically or systemically, were shown to inhibit or at least delay the development of form-deprivation myopia. Kearney et al. [39] were the first who demonstrated that similar relationships probably exist in humans, based on the observation that higher serum concentrations of Mel in the morning were associated with the occurrence of myopia in young adults.

Analyzing differences in the serum metabolic profiles of Chinese myopes and high myopes, Ke et al. [37] identified the citrate cycle as the most impacted pathway and postulated that this pathway might be involved in the axial length increase in humans. Citric acid intermediates in energy metabolism and corresponding elevated extracellular adenosine levels translate to greater adenosine receptor activation. This observation is supported by the results of animal experiments and findings from a few clinical trials; specifically, adenosine antagonist, 7-methylxanthine, was shown to reduce the eye elongation rate in form-deprivation myopia in macaque [63] and pigmented rabbit models [64], as well as in myopic children [65]. As circadian rhythms, or, more specifically, daylight exposure, are one of the most important modulators of adenosine levels in the mammalian retina, further research is needed to validate the importance of the findings mentioned above.

Liu et al. [38] reported on differences in the serum metabolomes of patients with choroidal neovascularization in the course of age-related macular degeneration, polypoidal choroidal vasculopathy and pathological myopia (PM). Thiamine metabolism, arginine and proline metabolism, as well as purine metabolism, were identified as the main contributors to CNV in PM development.

Most of the altered pathways identified in the study conducted by Du et al. [36] were related to oxidative stress (five pathways) and dopamine receptor D2 (five pathways), which provides a novel insight into the metabolic mechanisms involved in the occurrence, development and treatment of myopia.

Dai et al. [35] identified two metabolites, γ-glutamyltyrosine and 12-oxo-20-trihydroxy-leukotriene B4, as potential biomarkers of myopia. In turn, the metabolic alterations associated with high myopia included phospholipid, diacylglycerol, amino acid and vitamin metabolism.

### 4.3. Analytical Aspects

#### 4.3.1. Proteomics

The majority of proteomics studies are based on mass spectrometry combined with liquid chromatography (LC-MS). Most of these studies centered around the analysis of protein expression and interactions, protein post-translation modifications (PTMs) or enzymatic functions. Mass spectrometry enables us to track changes occurring throughout the proteome in various disease entities, including myopia. From a clinical trial perspective, a crucial aspect of proteomics analyses is quantitative information about the levels of proteins that differ significantly between the studied groups. Proteomic analyses based on mass spectrometry used in clinical trials can be divided into two categories. One of them is non-targeted analysis (data-dependent acquisition) (DDA) focusing on the use of metabolic labeling, chemical labeling or label-free protocols for the quantification of proteins or peptides. Another category is targeted analysis (data-independent acquisition) (DIA) based on the quantitative measurement of retention time and mass-to-charge with the MS equipment [66]. In the clinical aspect, most DDA strategies are based primarily on chemical labeling using isobaric tags—for example, tandem mass tags (TMT), isobaric tags for relative and absolute quantitation (iTRAQ) and others. Another method to quantify proteins and peptides in DDA analyses is label-free (LF) quantification. In this method, no chemicals are used for labeling, but quantitative information is obtained based on the measurement of the chromatographic peak’s area and integration with MS analysis [67]. Sequential window acquisition of all the theoretical ionic spectra (SWATH) is a method similar to LF. SWATH-MS is based on the cyclic acquisition of precursor ions with solid isolation windows that cover the entire m/z range and comparison of the spectra with the spectral library [68]. On the other hand, targeted proteomics uses two approaches: selected reaction monitoring (SRM) and parallel reaction monitoring (PRM). While in the PRM, the instrument records all peptide fragments from the analytical sample with a high mass resolution, only a single fragment ion is considered in the SRM [69].

Unlike in metabolomics studies, most proteomic studies included in this review were carried out on animal material. Animal specimens are easier to obtain than clinical samples, and the amount of analyte is larger. While proteomic analyses of human specimens were limited to vitreous [10,32] and aqueous humor [19,26,28], other materials, such as sclera [22,23], retina [17] and cornea [12], were also included in animal studies. Both studies in humans and those involving animal models can be divided into non-targeted and targeted analyses. As mentioned above, the non-targeted approach is characterized by different methods of protein and peptide quantification. Most of the studies included in this review used protein separation on polyacrylamide gel with electrophoresis, followed by protein digestion and mass spectrometry analysis. These methods are commonly used for proteomic analysis and protein separation. One of the two most widespread methods from this group is sodium dodecyl sulfate-polyacrylamide gel electrophoresis (SDS-PAGE). In SDS-PAGE, proteins are separated in a polyacrylamide gel based on their molecular weight [70]. The second method is the two-dimensional gel electrophoresis (2D-PAGE). In this method, proteins are separated based on their isoelectric point (pI) value in the first dimension, followed by the relative molecular weight-based separation in the second dimension [70]. The 2D-PAGE is quite often used in the preparation of tissue samples. Both SDS-PAGE and 2D-PAGE are commonly used in the examination of ocular tissues, with the latter employed somehow more often than the former [13,16,31]. However, both these methods represent quite an old approach to proteome analysis with relatively low throughput. With the progress in mass spectrometry, especially with the development of high-resolution mass spectrometers, this type of analysis is being abandoned, particularly in clinical trials. As mentioned above, an important aspect of clinical trials is a between-group comparison of protein levels. Thus, the methods for protein quantification have been used increasingly nowadays, with the most popular being the label-free technique and chemical labeling. The LF method was used, among others, to analyze the vitreous and retina from both humans [10,32] and animals [15,21,27]. In turn, chemical tags found application in the analysis of the proteomic profiles of the retina, vitreous and aqueous humor. Isotope-coded protein label (ICPL) was used in the studies of chick retina and vitreous humor [17,18], whereas iTRAQ was applied to analyze aqueous humor from patients with cataract [19,26] and atropine-treated mice with myopia [20]. While the latter analysis used the DDA approach, the SWATH analyses, especially those aimed at the creation of spectral libraries, involved the DIA approach. In most cases, the libraries were created based on vitreous and corneal samples from chicks [8,12] and retina from pigs [24,30]. Furthermore, this method was also used to analyze human tears in patients wearing orthokeratology lenses as myopia treatment [9]. The targeted approach, especially multiple reaction monitoring (MRM), was also used in the analysis of the retina from chicks with one myopic and one hyperopic eye [17]. Similar analyses were also performed on pig retina [24,30].

#### 4.3.2. Metabolomics

Metabolomics studies usually involve nuclear magnetic resonance (NMR) or mass spectrometry. NMR is suitable for the simultaneous identification and quantification of metabolites from different classes (e.g., amino acids, vitamins, thiols, carbohydrates), albeit in micromolar or higher concentrations [71]. MS has better sensitivity and dynamic range than NMR, but prior to the detection, metabolites usually need to be separated with liquid chromatography (LC-MS), gas chromatography (GC-MS) or capillary electrophoresis (CE-MS). Depending on the separation method, different classes of metabolites can be quantified. CE-MS is more suitable for polar and ionic, GC-MS for volatile and LC-MS for labile and non-volatile compounds. Consequently, to measure metabolites belonging to different classes and to increase the metabolome coverage, different separation methods need to be applied simultaneously [72]. Furthermore, it is noteworthy that the number of metabolites found in AH separation may differ depending on whether LC-MS, CE-MS or GC-MS was used as a separation method. For example, in one study [34], a total of 242 metabolites were initially identified in AH by means of GC-MS, with only 29 eventually found to be statistically significant. Meanwhile, in another study using CE-MS and LC-MS [33], the numbers of statistically significant AH metabolites exceeded 40 and 20, respectively. These discrepancies in the overall number of metabolites and the number of statistically significant metabolites are in part a consequence of the different statistical approaches used in various separation methods. In LC-MS and CE-MS, statistics are performed first, followed by the identification of distinctive metabolites. Meanwhile, the total number of distinctive metabolites is determined first in GC-MS, and then, those which are statistically significant are selected.

Regardless of the used method, different approaches to metabolome analysis exist. The most comprehensive approach is metabolic fingerprinting, the aim of which is to measure as many metabolites in the sample as possible. In this approach, all steps of the analytical protocol are optimized to facilitate the measurement of a large number of metabolites with appropriate quality [73]. Metabolic profiling methods focus on the measurement of metabolites from a particular class (e.g., fatty acids) [74] or metabolic pathway (e.g., arachidonic acid pathway) [75]. In MS-based fingerprinting studies, the presence of a reference group is necessary. The result is semi-quantitative, i.e., metabolites’ abundances between the controls and the case group are compared [73]. In contrast, metabolites determined by means of profiling or target analysis can be quantified, but respective analytical standards need to be used. The use of the standards is also indispensable to fully confirm the metabolite identification [76]. 

Only a few published studies have used metabolomic techniques to study myopia. To the best of our knowledge, none of the previous studies used NMR to study this condition. The vast majority of researchers applied untargeted methods, such as CE-MS [33], LC-MS [33,35,36] or GC-MS [34,37,38], to analyze human serum [35,36,37,38] or AH [33,34]. We found only one published study using a targeted method to analyze human serum [39] and only one untargeted study in an animal model [40].

The protocol of sample preparation for untargeted analysis depends on the type of examined material and chromatographic method applied. GC-MS differs considerably from the other two separation techniques in terms of sample preparation. A major limitation of GC-MS stems from the fact that this method is only capable of analyzing volatile compounds or those that can be made volatile by derivatization; this significantly extends and hinders the preparation procedure. Frequently, the samples are mixed with methanol [34,37] or methanol/chloroform mixture [38] and internal standard and then completely dried in a vacuum concentrator. Subsequently, the material is derivatized using a two-step procedure, methoxication and silylation. First, the samples are incubated with methoxyamine in pyridine, and then, BSTFA [34] or MSTFA [37,38] reagent containing 1% TMCS is added, and samples are again incubated. Finally, the samples are centrifuged, and the supernatants are transferred to vials and analyzed.

On the other hand, sample preparation for LC-MS involves mostly the addition of an organic solvent/solvent mixture to precipitate proteins and to extract metabolites. Barbas-Bernardos et al. used a minimal sample preparation protocol. For CE-MS analysis, the samples were mixed with the internal standard solution, and the material for LC-MS was only centrifuged, and then, the supernatant was analyzed directly [33]. The more complex preparation procedures described in the literature include deproteinization with methanol [35], methanol/chloroform or methanol/acetonitrile/water extraction buffer [36], followed by drying under nitrogen in most cases [35,36]. Then, the residues are dissolved and centrifuged, and the supernatants are used for further analyses [35].

In the only reported untargeted analysis based on an animal model, Yang et al. used GC-TOF-MS for retinal profiling in guinea pigs. The study required an extra step at the sample preparation stage; specifically, the samples of the retina were homogenized in a solvent mixture (chloroform/methanol/water) and centrifuged. Then, the supernatants were mixed with two internal standards and vacuum dried. The residues were derivatized as well [40].

In targeted research, the sample preparation procedure depends on the analyzed metabolite. Kearney et al. analyzed serum concentrations of dopamine and melatonin using liquid chromatography followed by on-line solid-phase extraction and tandem mass spectrometry analysis. In their study, the serum was only preserved at a final concentration of 0.1% ascorbic acid solution 0.1 M hydrochloric acid to prevent degradation of dopamine [39].

## 5. Conclusions

We propose to compare the AH, VH and serum metabolomic/proteomic profiles, especially from patients, where possible. Simultaneous metabolomics and proteomics analysis of these biological materials from the same patient might be of additional value, as it will enable a more holistic understanding of the disease. From the clinical perspective, the focus should be on the acquisition of biological material including serum, AH and VH from patients undergoing surgery. This might facilitate the discovery of new paths of myopia development as well as indicate new therapeutic targets. A combined approach using complementary separation techniques should also be considered. Especially in humans, studies on proteomics of serum and metabolomics of vitreous humor in myopia are lacking. Moreover, reports on metabolomics in animal models are scarce. Further studies on DA and mel–atropine interactions seem to be of particular importance from the clinical practitioner’s perspective.

## Figures and Tables

**Table 1:** Summary of published studies using proteomic analysis in myopia patients/animal models.

Type of Analytical Method	Type of Analysis	Type of Sample	Organism	Name of the Protein and/or Protein-Encoding Gene	Scientific Aspects	Ref.
SDS-PAGE MALDI TOF	untargeted	corneal epithelium	human	S100A4, KRT3, GSN, ENO1	comparison of patients with keratoconus vs. myopic patients as the controls	[7]
SWATH-MS	untargeted	vitreous humor	white Leghorn chicks	-	creation of a proteome library in emmetropization	[8]
SWATH-MS	untargeted	tears	human	-	creation of a proteome library in patients wearing orthokeratology lenses as treatment of myopia	[9]
Label-free LC-MS	untargeted	vitreous humor	human	PTGDS, GPX3	identification of expressed proteins in patients with pathological myopia and controls	[10]
2D-PAGE MALDI TOF	untargeted	sclera	shrews (Tupaia belangeri)	pigment epithelium-derived factor, procollagen Iα1, procollagen Iα2, thrombospondin I, glucose-regulated protein	analysis of differences in the development of lens-induced myopia and recovery from this condition	[11]
SWATH-MS	untargeted	cornea	white Leghorn chicks	-	creation of a corneal proteome library in high myopia	[12]
2D-PAGE MALDI TOF	untargeted	retina	mouse	Cryga, Cryba2, Cryba1	analysis of differences after exposure to various light conditions	[13]
2D-PAGE LC-MS	untargeted	retina, RPE, choroid	tilapia (Oreochromis niloticus)	annexin A5, gelsolin, TCP-1	analysis of differences in the protein profiles found in induced myopia	[14]
Label-free LC-MS	untargeted	vitreous humor	mouse	over 30 differentiating proteins	analysis of high myopia profiles with the low-density lipoprotein receptor-related protein 2	[15]
2D-PAGE MALDI-TOF	untargeted	retina	white Leghorn chicks	VIL1, DPYSL2, SARS, SEPTIN2, PGAM1 tubulin α, tubulin β2, tubulin α-chain, β-tubulin	analysis of differences in retinal proteins from lens-induced myopic chicks and controls	[16]
ICPL LC-MS, MRM LC-MS	untargeted, targeted	retina	white Leghorn chicks	VIM, APOA1, GSTM2	identification of proteins in myopic chicks and their association with excessive eye elongation	[17]
ICPL LC-MS	untargeted	vitreous humor	white Leghorn chicks	APOA1, TF, purpurin	identification of proteins differentiating myopia from hyperopia	[18]
iTRAQ LC-MS	untargeted	aqueous humor	human	ATP8A1, KRT2, KRT10, CRYAA, CRYBA4, CRYAA, CRYBB1, CRYBB2, CRYBA1, KRT6B, KRT6A, KRT14, KRT16	comparison of protein profiles in patients undergoing cataract surgery with concomitant myopia, glaucoma, or diabetes and controls	[19]
iTRAQ LC-MS	untargeted	retina	mouse	over 25 differentiating proteins	analysis of atropine effect on retina proteome in myopic mice	[20]
Label-free LC-MS	untargeted	retina	chicks	analysis of a biochemical pathway	identification of pathways involved in myopia and hyperopia	[21]
2D-PAGE MALDI-TOF	untargeted	sclera	guinea pig	Cryab, CryaA	analysis of changes in protein profiles during the development of form-deprivation myopia and recovery from this condition	[22]
2D-PAGE LC-MS	untargeted	sclera	tree shrew	over 50 differentiating proteins	analysis of changes in the protein profiles of lens-induced myopia and recovery from this condition	[23]
SWATH, MRM-MS	untargeted, targeted	retina	guinea pigs	-	creation of a proteome library in emmetropization	[24]
2D-PAGE MALDI-TOF	untargeted	retina and fibrous sclera	chicks	APOA1, CRMP-62, CKB, ENO2, tubulin α-1 chain, VIM	study of emmetropization	[25]
iTRAQ LC-MS	untargeted	aqueous humor	human	over 200 differentiating proteins	identification of proteins contributing to the development of cataract in myopic patients	[26]
Label-free LC-MS	untargeted	retina, retinal pigment epithelium	chicks (White Leghorn/New Hampshire)	over 65 differentiating proteins	analysis of proteomic responses to early optical defocus in relation to transcriptome-level changes	[27]
2D-PAGE MALDI-TOF	untargeted	aqueous humor	human	ALB, TTR, GC	comparison of the proteome in high myopia patients and controls	[28]
2D-PAGE MALDI-TOF	untargeted	retina	guinea pig	ACTB, MDH1, Rab-11B, PKM2, ACP1	analysis of differential protein expression in response to lens-induced myopia	[29]
SWATH-MS, MRM-MS	untargeted targeted	retina	guinea pig	-	creation of a spectral library of protein profile changes during emmetropization	[30]
2D-PAGE LC-MS	untargeted	retina	chick	ARR3, Rab-11B, PSMD14, β-tubulin, PRDX6, UCH-L1	proteome study during early recovery from lens-induced myopia	[31]
Label-free LC-MS	untargeted	vitreous humor	human	over 50 differentiating proteins	analysis of protein expression profiles in vitreous humor from patients with pathologic myopic retinoschisis with/without intravitreal antivascular endothelial growth factor therapy	[32]

SWATH: sequential window acquisition of all theoretical fragment ion spectra; RPE: retinal pigment epithelium; ICPL: Isotope-coded protein label; MRM: multiple reaction monitoring; iTRAQ: isobaric tags for relative and absolute quantitation.

**Table 2:** Summary of published studies using metabolomic analysis in myopia patients/animal models.

Type of Analytical Method	Type of Analysis	Type of Sample	Organism	Type of Analytical Method	Potential Biomarkers or Altered Pathways	Ref.
CE-TOF-MS	untargeted	aqueous humor	human	comparison of patients with high myopia (*n* = 12) and low myopia (*n* = 24)	aminooctanoic acid, L-arginine, citrulline, aminoundecanoic acid, L-cysteinylglycine disulfide	[33]
LC-QTOF-MS	untargeted	aqueous humor	human	comparison of patients with high myopia (*n* = 12) and low myopia (*n* = 24)	trihydroxyphenyl-gamma-valerolactone, dihydropteroic acid, dodecanedioic acid, aminocyclohexanecarboxylic acid, butyryl-L-carnitine, pantothenic acid, didehydro-retinoic acid, sphinganine, histidinyl-phenylalanine, dimethylnonanoyl carnitine, PC(O-32:2)//PC(P-32:1), PC (42:6), C24 sulfatide, PC(P-42:2)//PC(O-42:3), LacCer(d40:0), trihexosylceramide (d36:2), NeuAcaGalCer(d42:2)	[33]
GC-TOF-MS	untargeted	aqueous humor	human	metabolic profiling in patients with high myopia (*n* = 20) and controls (*n* = 20)	glutamine, N-alpha-acetyl-L-ornithine, nicotinoylglycine, oxalacetic acid, o-hydroxyhippuric acid, oxalic acid, ribose, cis-gondoic acid, linoleic acid methyl ester, thymidine, phosphate, indole-3-acetamide, 2-aminophenol, 2-ketoadipate, 3-phenyllactic acid, cis-phytol, conduritol b epoxide, salicin	[34]
LC-QTOF-MS	untargeted	serum	human	metabolic profiling in high myopia cases (*n* = 30) and controls (*n* = 30)	γ-glutamyltyrosine and 12-oxo-20-trihydroxy-leukotriene B4	[35]
UHPLC-MS	untargeted	serum	human	metabolomics profiling in myopia cases (*n* = 108) and controls (*n* = 103)	steroid biosynthesis, lysine degradation, arginine and proline metabolism, glycerolipid metabolism, glycerophospholipid metabolism, arachidonic acid metabolism, linoleic acid metabolism, sphingolipid metabolism	[36]
UHPLC-MS	untargeted	serum	human	lipid profiling in myopia cases (*n* = 108) and controls (*n* = 103)	steroid biosynthesis, lysine degradation, glycerolipid metabolism, glycerophospholipid metabolism, arachidonic acid metabolism, linoleic acid metabolism, alpha-linolenic acid metabolism, sphingolipid metabolism	[36]
GC-TOF-MS	untargeted	serum	human	metabolomic analysis of patients with high myopia (*n* = 40) and low myopia (*n* = 40)	alanine, mannose, itaconic acid, aconitic acid, O-acetylserine, phthalic acid, abietic acid, salicin, citric acid, aminomalonic acid, palmitoleic acid, conduritol b epoxide, shikimic acid, 4-hydroxyphenylacetic acid, hesperitin, anandamide, oxalacetic acid, pimelic acid, 2-ketoadipate, N-ethylmaleamic acid	[37]
GC-TOF-MS	untargeted	serum	human	metabolic profiling in patients with pathological myopia (*n* = 57) and controls (*n* = 81)	hypoxanthine, L-2-amino-3-(1-pyrazolyl)propanoic acid, linoleic acid, maleic acid, ribonolactone	[38]
LC-On-Line SPE-MS/MS	targeted	serum	human	myopia patients (*n* = 25) and controls (*n* = 29) at the baseline and after 18-month follow-up (22 patients and 23 controls)	melatonin, dopamine	[39]
GC-TOF-MS	untargeted	retina	guinea pig	time-dependent form-deprivation myopia, T = 3 days, 12 cases and 5 controls	mannose, urea, glucose, arabinose, tyrosine, glutamic acid, threonine, valine, isoleucine, malic acid, alanine	[40]
GC-TOF-MS	untargeted	retina	guinea pig	time-dependent form-deprivation myopia, T = 2 weeks, 12 cases and 6 controls	threonine, valine, isoleucine, malic acid, alanine, arachidic acid (20:0), octadecenoic acid (18:1), octadecanoic acid (18:0), arachidonic acid (20:4), cholesterol, ethanolamine, hexadecanoic acid (16:0), tetradecanoic acid (14:0), octadecadienoic acid (18:2), 2-ketoglutaric acid, GABA	[40]

Numbers in brackets are the numbers of patients included in the research.

**Table 3:** Metabolic pathways corresponding to metabolites identified in serum samples.

Pathway	No. of Metabolites in the Pathway	No. of Metabolites Detected in Serum	*p*-Value	Pathway Impact
**Sphingolipid metabolism**	21	8	0.00013	0.57
**Citrate cycle (TCA cycle)**	20	5	0.019	0.33
Linoleic acid metabolism	5	2	0.056	1.0
Biosynthesis of unsaturated fatty acids	36	6	0.068	0.0
**Alanine, aspartate and glutamate metabolism**	28	5	0.072	0.094
**Tryptophan metabolism**	41	6	0.11	0.17
**Glyoxylate and dicarboxylate metabolism**	32	5	0.11	0.12
Tyrosine metabolism	42	6	0.12	0.19
Glycerolipid metabolism	16	3	0.14	0.29
Retinol metabolism	17	3	0.16	0.46
Glycerophospholipid metabolism	36	5	0.17	0.35
**Pyruvate metabolism**	22	3	0.27	0.21
Glycine, serine and threonine metabolism	33	4	0.28	0.26
**Lysine degradation**	25	3	0.34	0.14
Thiamine metabolism	7	1	0.45	0.0
Ascorbate and aldarate metabolism	8	1	0.50	0.0
Taurine and hypotaurine metabolism	8	1	0.50	0.43
Cysteine and methionine metabolism	33	3	0.52	0.021
Beta-alanine metabolism	21	2	0.52	0.45
One carbon pool by folate	9	1	0.54	0.0
Steroid hormone biosynthesis	85	7	0.56	0.023
Propanoate metabolism	23	2	0.57	0.041
Phenylalanine metabolism	10	1	0.57	0.0
Biotin metabolism	10	1	0.57	0.15
**Arginine and proline metabolism**	38	3	0.61	0.012
**Glycolysis or Gluconeogenesis**	26	2	0.64	0.10
Galactose metabolism	27	2	0.66	0.0
Alpha-linolenic acid metabolism	13	1	0.67	0.0
Glutathione metabolism	28	2	0.68	0.023
**Arginine biosynthesis**	14	1	0.70	0.0
Glycosylphosphatidylinositol(GPI)-anchor biosynthesis	14	1	0.70	0.0040
Histidine metabolism	16	1	0.75	0.0
Starch and sucrose metabolism	18	1	0.79	0.073
Terpenoid backbone biosynthesis	18	1	0.79	0.18
**Pantothenate and CoA biosynthesis**	19	1	0.80	0.021
Arachidonic acid metabolism	36	2	0.81	0.0
Fructose and mannose metabolism	20	1	0.82	0.0
Selenocompound metabolism	20	1	0.82	0.0
Ether lipid metabolism	20	1	0.82	0.0
Amino sugar and nucleotide sugar metabolism	37	2	0.82	0.0058
**Pyrimidine metabolism**	39	2	0.84	0.057
Steroid biosynthesis	42	2	0.87	0.0
**Aminoacyl-tRNA biosynthesis**	48	2	0.92	0.17
Drug metabolism - cytochrome P450	55	2	0.95	0.0
Fatty acid elongation	39	1	0.96	0.0
Fatty acid degradation	39	1	0.96	0.0
**Purine metabolism**	65	2	0.97	0.022
Primary bile acid biosynthesis	46	1	0.98	0.0076
Fatty acid biosynthesis	47	1	0.98	0.015

Pathway impact values and *p*-values were obtained from metabolic pathway analysis performed with MetaboAnalyst 4.0 software. Pathways highlighted in bold were identified in the case of both serum and AH samples.

**Table 4:** Metabolic pathways corresponding to metabolites identified in AH samples.

Pathway	No. of Metabolites in the Pathway	No. of Metabolites Detected in AH	*p*-Value	Pathway Impact
**Arginine biosynthesis**	14	4	0.000028	0.30
**Alanine, aspartate and glutamate metabolism**	28	2	0.058	0.21
**Glyoxylate and dicarboxylate metabolism**	32	2	0.074	0.024
D-Glutamine and D-glutamate metabolism	6	1	0.082	0.0
Nitrogen metabolism	6	1	0.082	0.0
**Pyrimidine metabolism**	39	2	0.10	0.06
**Aminoacyl-tRNA biosynthesis**	48	2	0.15	0.0
**Pantothenate and CoA biosynthesis**	19	1	0.24	0.0071
**Citrate cycle (TCA cycle)**	20	1	0.25	0.12
**Sphingolipid metabolism**	21	1	0.26	0.15
Pentose phosphate pathway	22	1	0.27	0.0
**Pyruvate metabolism**	22	1	0.27	0.0016
**Lysine degradation**	25	1	0.30	0.14
**Glycolysis or Gluconeogenesis**	26	1	0.31	0.0
Folate biosynthesis	27	1	0.32	0.0
**Arginine and proline metabolism**	38	1	0.42	0.056
**Tryptophan metabolism**	41	1	0.45	0.0
**Purine metabolism**	65	1	0.61	0.0

Pathway impact values and *p*-values were obtained from metabolic pathway analysis performed with MetaboAnalyst 4.0 software. Pathways highlighted in bold were identified in the case of both serum and AH samples.

## Abbreviations

2D-PAGE	two-dimensional gel electrophoresis
AH	aqueous humor
CE	capillary electrophoresis
DDA	data-dependent acquisition
DIA	data-independent acquisition
ERM	epiretinal membrane
FDM	form-deprived myopia
VH	vitreous humor
GC	gas chromatography
ICPL	isotope-coded protein label
iTRAQ	isobaric tags for relative and absolute quantitation
HM	high myopia
MH	macular hole
LC	liquid chromatography
LIM	lens-induced myopia
LF	label-free
MS	mass spectrometry
MALDI-TOF	matrix-assisted laser desorption/ionization
MRS	myopic retinoschisis
MRM	multiple reaction monitoring
NMR	nuclear magnetic resonance
PCV	polypoidal choroidal vasculopathy
PRM	parallel reaction monitoring
PM	pathological myopia
PPV	pars plana vitrectomy
PTM	post-translation modifications
RPE	retinal pigment epithelium
RRD	rhegmatogenous retinal detachment
SRM	selected reaction monitoring
SER	spherical equivalent refraction
SDS-PAGE	sodium dodecyl sulfate-polyacrylamide gel electrophoresis
SWATH	sequential window acquisition of all theoretical fragment ion spectra
TMT	tandem mass tags
